# Be(ing) prepared: Guide and Scout participation, childhood social position and mental health at age 50—a prospective birth cohort study

**DOI:** 10.1136/jech-2016-207898

**Published:** 2016-11-10

**Authors:** Chris Dibben, Chris Playford, Richard Mitchell

**Affiliations:** 1 University of Edinburgh, Institute of Geography, Edinburgh, UK; 2 University of Edinburgh, Administrative Data Research Centre Scotland, Edinburgh, UK; 3 Department Public Health, University of Glasgow, Glasgow, UK

**Keywords:** Life course epidemiology, MENTAL HEALTH, DEPRESSION, PHYSICAL ACTIVITY, INEQUALITIES

## Abstract

**Background:**

Mental health is a major concern in many countries. We explore whether youth participation in the Scouts and Guides could protect mental health in later life and in particular whether it might reduce inequalities in mental health associated with early life socioeconomic position.

**Methods:**

Using the 1958 birth cohort National Child Development Study, we tested whether Scouts–Guide attendance was associated with mental health (SF-36, Mental Health Index (MHI-5)) controlling for childhood risk factors and interacted with social class.

**Results:**

Of the 9603 cohort members, 28% had participated in the Scouts–Guides. The average MHI-5 score was 74.8 (SD 18.2) at age 50. After adjustment, for potential childhood confounders, participation in Scouts–Guides was associated with a better MHI-5 score of 2.22 (CI 1.32 to 3.08). Among those who had not been a Scout–Guide, there was a gradient in mental health at age 50 by childhood social position, adjusting for other childhood risk factors. This gradient was absent among those who had been a Scout–Guide. Scout–Guides had an 18% lower odds of an MHI-5 score indicative of mood or anxiety disorder. The findings appeared robust to various tests for residual confounding.

**Conclusions:**

Participation in Guides or Scouts was associated with better mental health and narrower mental health inequalities, at age 50. This suggests that youth programmes that support resilience and social mobility through developing the potential for continued progressive self-education, ‘soft’ non-cognitive skills, self-reliance, collaboration and activities in natural environments may be protective of mental health in adulthood.

## Introduction

Mental health is a major concern in many countries, with a high cost to society and individuals, particularly in middle age^1^ when socioeconomic health inequalities are at their greatest.^2^ ^3^ The considerable amounts of primary care time spent addressing mental health issues and increasing expenditure on mental health prescribing^4^ mean effective preventive interventions are important. There is also evidence that an adverse socioeconomic situation in childhood has deleterious effects on mental health in later life and social advantage is protective.^5^ ^6^ What can be done to improve mental health, and reduce the long-term impacts of socioeconomic adversity in childhood?

Programmes which enable an individual to develop capabilities and resilience are known to have a positive effect on outcomes in youth,^7^ particularly those focused on ‘positive youth development’^8^ aimed at developing: social, vocational and cognitive competence; self-confidence; connection to others; respect for societal and cultural rules and caring and compassion.^9^ It is plausible that the skills, capabilities and resilience acquired in youth development schemes will also be protective of later life mental health, but evidence about the persistence of benefits from these programme is almost entirely absent. We hypothesised that exposure to such programmes over the long-term may have a lasting effect. Crucially however, we also hypothesised that, for children from less advantaged backgrounds at home and school, the abilities and experience gained in this kind of youth development programme would be particularly important and might provide access to skills that their peers acquire as part of their advantaged position. In order to understand the potential long-term impact of structured youth programmes, we carried out a prospective cohort study of later life mental health of individuals (aged 50) who had been Scouts–Guides in the 1970s, a movement whose methods, at that time, were close to those identified as key to ‘positive youth development’^10^ and, though operationalised through local organisations, were managed, to ensure consistency, through detailed programmes and standards governed by regional oversight.^11^ ^12^ We ask whether:

1. Childhood participation in Scouts–Guides is independently associated with better mental health at age 50?
2. Childhood participation in Scouts–Guides reduce inequalities in adult mental health associated with early life socioeconomic position?

## Methods

### Study design and population

We used the National Child Development Study (NCDS), an ongoing cohort study of people born in the UK in a single week in 1958^13^ (n∼17 500). We used the information on the 9790 study members who were interviewed age 50 in 2008 (the most recent measurement of mental health). Analysis of attrition in the NCDS (by age 45) suggests that these study members are broadly representative of the surviving cohort.^14^ Although the NCDS data would support a prospective cohort design assessment of effect of Scout–Guide attendance in childhood on mental health in later life, there are multiple threats to the validity of such an approach.^15^ We were particularly concerned about factors affecting a young person's probability of attending Scouts–Guides, which could also be protective of mental health in later life. To test for this possibility, we used three approaches. First, we explored a form of ‘negative control’ exposure.^16^ Specifically, we investigated whether attendance at other types of organisations (voluntary groups and the church), and other outside school clubs, which might have a similar set of common factors encouraging attendance *and* which might be protective of mental health in later life, were also associated with better later life mental health, conditioning on scouts–guides attendance. The absence of an association would suggest that an unmeasured *common* factor was not confounding the scout–guide association, while the existence of an association would be ambiguous with an unmeasured common factor and a third factor, independent of scout–guide attendance, but acting in a pathway through the other types of organisation on mental health, being possible. Second, we sought evidence of a dose–response relationship, exploring whether fractions of attendance were related to better mental health. Third, we explored whether the Scout–Guide association was weaker in regions with a higher proportional attendance which might suggest Scout–Guides not being the causative agent. Collectively, these strategies provided a strong test of whether residual confounding or inadequate control (through measurement error) affected the study results.

### Outcome measure

The Mental Health Index (MHI-5) of the SF-36 item questionnaire is a well-validated instrument comprising five items, representing four major mental health dimensions.^17^ ^18^ These items ask about the amount of time during the past 4 weeks a person has ‘(1) been a very nervous person?; (2) felt calm and cheerful?; (3) felt downhearted and low?; (4) been a happy person?; and (5) felt so down in the dumps that nothing could cheer you up?’. Items are combined to form a scale varying between 0 and 100, with higher scores indicating better mental health. An indicator of potential mood or anxiety disorders was derived using a cut-off score of 65 points or less.^19^

### Variables

Respondents were asked aged 50 whether they had/were a member of: Scouts–Guides, religious group or church organisation, or voluntary service group. We used variables collected at the appropriate sweeps of the NCDS to control for childhood factors which might affect Scout–Guide participation and later life mental health. These were sex, family social class, familial history of mental health and family aspiration for the child. Social position was measured using the Cambridge Social Interaction and Stratification (CAMSIS) scale^20^ and Register General's Social Class (RGSC). Family history of mental illness was asked age 7, specifically focusing on ‘Neurosis’. Family aspiration was estimated from parents' desire that their child leaves school at the minimum age, asked at age 11. A question asked aged 11 on the ‘frequency pupil goes to clubs outside school’ was used to estimate attendance and participation in other types of clubs but not Scout–Guides. The aggregated proportion of cohort members in a Government Office Region who were in the Scouts–Guides in 1974 was calculated and centred by subtracting the UK mean participation rate. Other childhood behaviours were captured by questions on^1^ how often they played indoor games or sport at age 16, and^2^ played outdoor games or sport at age 16.

### Statistical analyses

Of the ∼17 500 initial study members, some 9790 were interviewed in 2008. Of these, only 4020 had complete cases. Multiple imputation (MI) using chained equations was used to impute values for the missing data, with the data assuming to be missing at random (MAR), given covariates predicting missingness. Ten imputations were run separately for those who had or not been a Scout–Guide. The patterns of missingness and MI model are described fully in the online supplementary appendix. Of the 9790 cases available, 187 were excluded (105 were current Scout–Guides and 82 were missing on variables used within the MI model). This represents a minor loss of information (1.2% of cases) but enabled a more parsimonious MI model to be estimated.

10.1136/jech-2016-207898.supp1 Supplementary data

The analysis presented in this paper is based on MI data sets. A complete case analysis was also carried out. Stata V.13 was used (Stata Statistical Software: Release V.13 [program]. College Station, Texas: StataCorp LP, 2013.), with the MHI-5 score modelled using ordinary least squares (OLS) regression reporting β-coefficients, SEs and model fit (R^2^). The unadjusted OLS estimate for the association between being a Scout–Guide and MHI-5 score was compared with estimates adjusting for childhood confounders, and for regional participation rate in Scout–Guides. The sensitivity of the analysis to non-normality in the MHI-5 score was tested through replication with a suitable transformation and is shown in the online supplementary materials. The probability of mood or anxiety disorders (based on an MHI-5 score of <65) was modelled using logistic regression adjusting for childhood confounders with ORs and 95% CIs reported. p Values indicting the level of statistical significance are reported with asterisks. Adjusted predicted values at mean values of MHI-5 score were estimated based on OLS models, including interaction terms between father's CAMSIS and Scout–Guide membership, and father's RGSC and Scout–Guide membership.

## Results

Of the 9603 cases, 28% had been Scouts–Guides (table 1). There was little variation in attendance by parental social position. Participation in Scouts–Guides varied across Britain, being less likely for those who lived in Wales during in childhood, and more likely in for those who lived in Scotland. Few parents of cohort members, asked in 1969, hoped their children would leave school at the minimum age (5.3%). This response was far less likely (2.8%) among those whose children were attending Scouts–Guides. Those who were Scouts–Guides were also 2–3 times as likely to have been, or still be, a member of church or voluntary groups. Attendance was not related to familial history of mental illness.

**Table 1:** Descriptive statistics for sample (n=9603)

	Scout/Guide participation (2008)			
	Never	Previous	Total	
	% (Col.)	% (Col.)	% (Col.)	Test for difference in proportion p value
Scout/guide participation				
Never			72.4	
Previous			27.6	
Voluntary group participation				
Never	90.6	83.3	88.6	<0.01
Previous	5.5	11.6	7.2	<0.01
Current	3.9	5.1	4.2	0.01
Church participation				
Never	87.3	73.9	83.6	<0.01
Previous	5.4	14.5	7.9	<0.01
Current	7.3	11.6	8.5	<0.01
Sex				
Female	50.3	51.7	50.7	0.24
Male	49.7	48.3	49.3	0.24
RGSC: Social class of Mother's Husband (GRO 1951)				
Social class I	4.6	5.9	5.0	0.01
Social class II	12.9	17.4	14.1	<0.01
Social class III non-manual	9.8	11.9	10.4	0.01
Social class III manual	50.4	49.7	50.2	0.57
Social class IV	12.9	9.0	11.8	<0.01
Social class V	9.4	6.0	8.4	<0.01
Family difficulties—mental illness (1965)				
No	89.2	90.4	89.5	0.13
Do not know	8.0	7.0	7.7	0.12
Yes	2.8	2.6	2.8	0.71
Parental hopes child's school leaving (1969)				
Leave min age	5.3	2.8	4.6	<0.01
Stay on longer	74.7	84.0	77.2	<0.01
Do not know yet	20.0	13.3	18.1	<0.01
Pupil goes to clubs outside school (1969)				
Most days	19.6	43.8	26.3	<0.01
Sometimes	21.7	28.0	23.4	<0.01
Hardly ever	58.7	28.2	50.2	<0.01
Government Office Region at age 16				
North	7.5	5.6	7.0	<0.01
North West	11.6	12.5	11.8	0.24
East and West Riding of Yorkshire	9.0	5.9	8.2	<0.01
North Midlands	7.8	7.2	7.7	0.29
Midlands	10.1	8.4	9.7	0.01
East	9.2	8.5	9.0	0.30
South East	17.1	18.7	17.5	0.08
South	6.4	7.3	6.7	0.16
South West	6.9	6.7	6.9	0.64
Wales	6.3	3.8	5.6	<0.01
Scotland	7.9	15.5	10.0	<0.01
How often plays outdoor games and sport (1974)				
Often	36.5	39.9	37.4	0.01
Sometimes	35.9	35.9	35.9	0.98
Hardly ever	24.5	21.2	23.6	<0.01
No chance	3.1	3.0	3.1	0.77
How often plays indoor games and sport (1974)				
Often	24.4	27.0	25.1	0.03
Sometimes	32.7	32.0	32.5	0.61
Hardly ever	32.9	29.2	31.9	<0.01
No chance	10.0	11.8	10.5	0.02

Table 2 shows results from linear modelling of the MHI-5 score. Model 1 shows a mean mental health score of 74.0 for non-Scouts–Guides (range 0–100). Past participation in Scout–Guides was associated with a 2.7 point higher score. In model 2, controlling for the childhood risk factors listed in the table title, the Scout–Guide association was reduced slightly to 2.2. Scout–Guide attendance among cohort members in the 1970s varied regionally (table 1). In model 3, we allowed the Scout–Guide mental health association to vary by regional participation rate. The Scout–Guide association was strengthened slightly (to 2.3 points) and this model explained 3% of the variance in the mental health score. Scouts–Guides association was not notably weaker in areas with high participation. Unless some unmeasured variables were influencing participation rate and mental health, this suggests the absence of residual confounding. Attendance of either scouts (male only model 4) or guides (female only model 5) had a similar association with mental health.

**Table 2:** Linear regression models for Short-Form Health Survey (SF-36) mental health index

	Model 1		Model 2		Model 3		Model 4, male		Model 5, female	
	B	SE	B	SE	B	SE	B	SE	B	SE
Scout/guide										
Never	0	0	0	0	0	0	0	0	0	0
Previous	2.68***	(0.45)	2.22***	(0.45)	2.28***	(0.46)	2.38***	(0.62)	2.08***	(0.62)
Government Office Region participation (%)					0.015	(0.039)				
Not scout/guide by GOR					0	0				
Scout/guide by GOR					−0.065	(0.066)				
Voluntary group										
Never			0	0	0	0	0	0	0	0
Previous			−1.98**	(0.76)	−1.99**	(0.76)	−3.31**	(1.24)	−1.22	(0.97)
Current			0.97	(0.96)	0.95	(0.96)	0.89	(1.47)	1.04	(1.28)
Church group										
Never			0	0	0	0	0	0	0	0
Previous			−0.60	(0.74)	−0.59	(0.74)	−1.06	(1.13)	−0.21	(1.00)
Current			−0.18	(0.70)	−0.16	(0.70)	−0.18	(1.11)	−0.12	(0.91)
Constant	74.0***	(0.23)	68.4***	(1.61)	68.4***	(1.62)	71.7***	(2.83)	66.8***	(2.14)
Observations	9603		9603		9603		4733		4870	
R^2^	0.004		0.03		0.03		0.03		0.03	

(MHI-5) score (aged 50) adjusting for (in models 2–5): Father's CAMSIS score, Sex, Family Difficulties—Mental Illness (1965), Parental hopes child's school leaving (1969), How often plays outdoor games and sport (1974), How often plays indoor games and sport (1974).

SE in parentheses, *p<0.05, **p<0.01, ***p<0.001.

Previous or current participation in voluntary and church groups was not associated with better mental health in any of the three models (tables 2 and 3). This suggests that there was not a common unmeasured variable affecting attendance of all these organisations and mental health, confounding the main finding.

**Table 3:** Odds of a mood or anxiety disorder (score of <65 on Short Form Health Survey (SF-36) Mental Health Index (MHI-5)) adjusting for: Father's Register Generals Social Class score, Sex, Family Difficulties—Mental Illness (1965), Parental hopes child's school leaving (1969), How often plays outdoor games and sport (1974), How often plays indoor games and sport (1974)

	Model 4	
	OR	95% CI
Scout/guide		
Never	1.00	
Previous	0.82***	(0.74 to 0.92)
Voluntary group		
Never	1.00	
Previous	1.27**	(1.06 to 1.52)
Current	1.00	(0.78 to 1.27)
Church group		
Never	1.00	
Previous	1.03	(0.86 to 1.23)
Current	0.99	(0.83 to 1.18)
Observations	9603	

Exponentiated coefficients; 95% CIs in parentheses, *p<0.05, **p<0.01, ***p<0.001.

Table 3 shows the odds of having a mood or anxiety disorder, controlling for the childhood variables. Scouts–guides had an 18% lower odds of an MHI-5 score indicative of mood or anxiety disorder.

**Figure 1:**
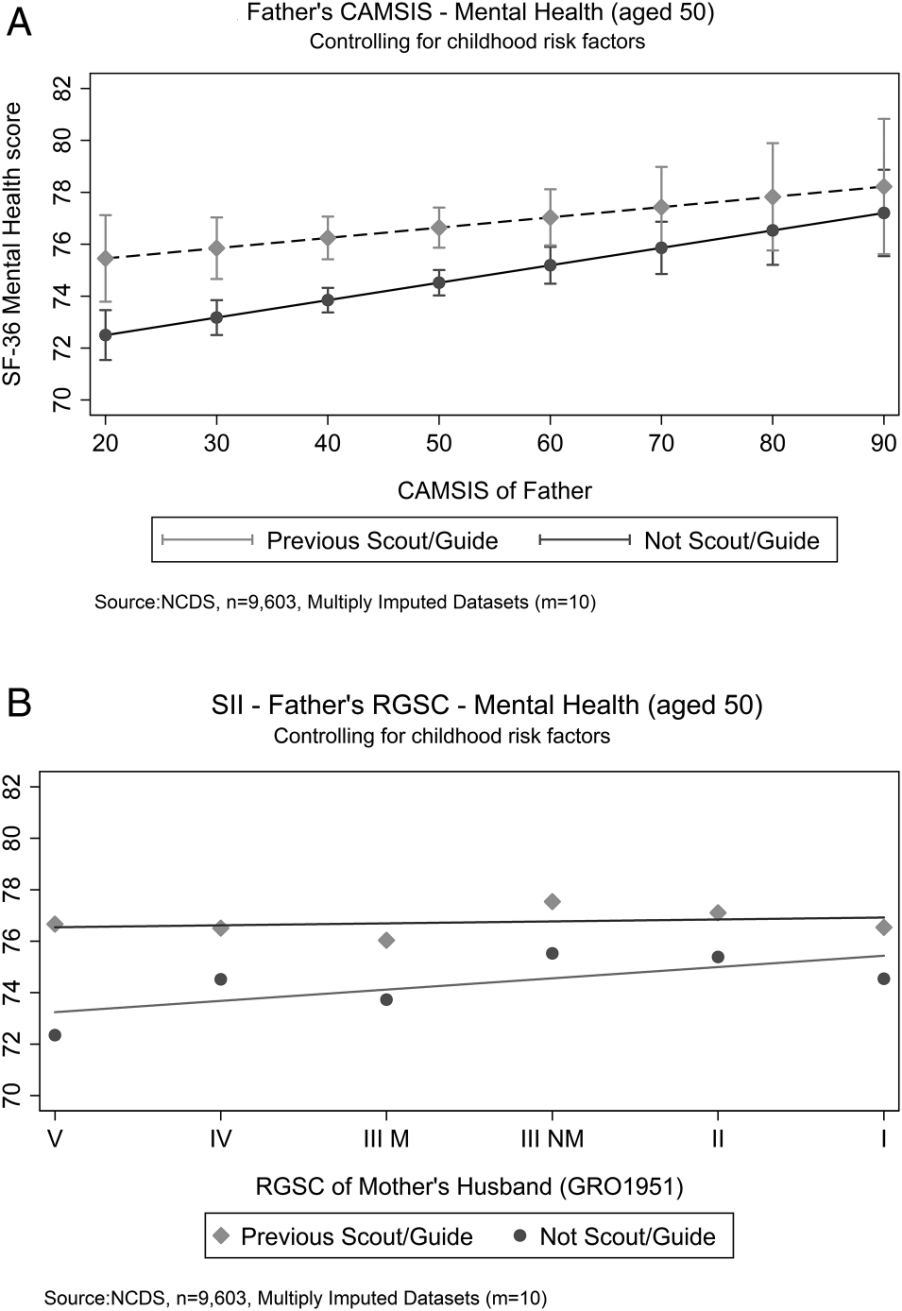
Father's Cambridge Social Interaction and Stratification/Register General's Social Class relationship with Mental Health (aged 50) adjusting for in (A) and (B): Voluntary Group Participation, Church Participation, Sex, Family Difficulties—Mental Illness (1965), Parental hopes child's school leaving (1969), How often plays outdoor games and sport (1974), How often plays indoor games and sport (1974).

The adjusted predictions at the means, for the models assessing interaction between childhood socioeconomic position and Scout–Guide attendance, are shown in figure 1A for individuals by their father's CAMSIS score (score 1–100 (highest social position)) and in figure 1B for individuals by their father's RGSC with inequality slope fitted. There was a strong gradient in mental health by childhood social position for those who had not been a Scout–Guide, adjusting for other childhood risk factors (figure 1A, B). Lower social position was associated with ∼3 points worse mental health score in figure 1A (slope, p<0.001). In contrast, there appeared to be no or at least a weaker (figure 1A) (slope, p=0.172) socioeconomic gradient for those who had been a Scout–Guide.

**Figure 2:**
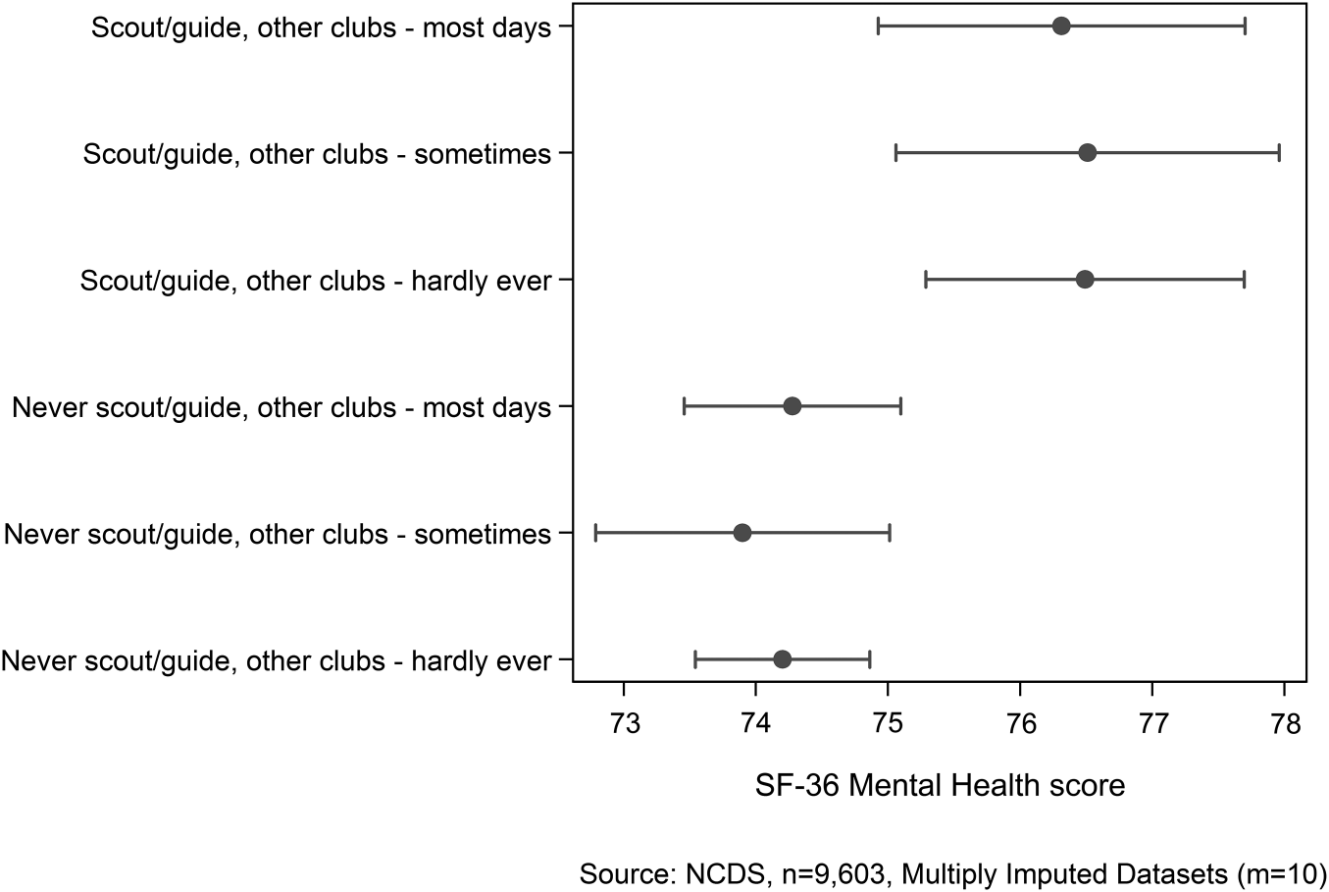
Estimate of ‘dose–response’ from level of Scout–Guide attendance on mental health adjusting for: Register General's Social Class of Mother's Husband, Voluntary Group Participation, Church Participation, Sex, Family Difficulties—Mental Illness (1965), Parental hopes child's school leaving (1969), How often plays outdoor games and sport (1974), How often plays indoor games and sport (1974).

Figure 2 shows the mental health score associated with the estimated frequency of attendance at out of school clubs at age 11. There is no evidence of a dose–response relationship based on estimating scout–guide attendance from a question on attendance of outside school clubs at age 11. However, the difference between those who were not Scouts–Guides, but attended other outside school clubs most days, and those who attended Scouts–Guides was not attenuated compared with the overall Scout–Guide effect of 2.22 (for model 2). This suggests that although there might be a common unmeasured early life effect making attendance at any type of outside school clubs more likely, while also being protective of later life mental health, it does not explain the Scout–Guide mental health association, otherwise there would be no difference in mental health between these two groups.

## Discussion

In this prospective cohort study, we found evidence that having been a member of the Guides or Scouts in the 1970s was associated with better mental health at age 50. Even when controlling for early life factors, young people who were members of the Scouts–Guides have better mental health in later life than their non-attending peers. Perhaps most strikingly, cohort members who had been a Scout–Guide had an 18% lower odds of a mood or anxiety disorder at age 50, controlling for childhood factors, or a rate of around 210 per 1000 in the Scout–Guide exposed population rather than 250 per 1000 for those not exposed. Childhood participation in Scouts–Guides also appears to reduce perhaps even remove inequalities in adult mental health associated with early life socioeconomic position. This suggests that Scout–Guide attendance may be protective, instituting a resilience to stressful life events that may lead to mental ill health or increase the chances of achieving states in adult life that are associated with better mental health. The relationship does not appear to be explained by potential confounding factors, notably familiar characteristics such as parental attitudes to education or familial history of mental health which might have affected the likelihood of a child being a Scout–Guide and mental health in later life. Our findings support those of the one other study that we are aware of, that has looked at the relationship between having been Scouts and mental health.^21^

### Possible explanations and implications

Many of the elements that make up the Scout–Guide approach to youth development are implicated in better adult mental health. It is suggested, for example, that there are benefits for mental health from a range of lifestyle factors, including exercise, nutrition and diet, contact with nature and the outdoors, positive social relationships, recreation, relaxation and stress management, religious and spiritual involvement and contribution and service.^22^ During the 1970s, the Guide ‘method’ had evolved into an ‘eight point plan’, aimed at developing not only the traditional ideas of: character, out-of-doors skills and challenges, fitness, homecraft skills and service, but also creative ability, mind and relationships, and a focus on personal development then rather simply character development.^11^ Similarly, the Scouts were focusing on a system of progressive self-education based on: promises (laws), active learning, interactions within small groups and stimulating individual-driven self-learning through awards.^12^

The movement's methods were based on a system of progressive self-education taking place in the context of small peer groups with adults guiding the process.^11^ ^23^ Not being purely recreational and unstructured, as a youth sport club might be,^24^ it used activities to allow young people to learn ‘to know’, ‘to be’, ‘to do’ with adults assisting, rather than directing. Perhaps, this set of ‘capabilities’ for continued self-learning enable the participants to structure and run their adult lives in a way that is relatively more protective against mental ill health. It may also be that these activities develop ‘soft’ non-cognitive skills, such as confidence, personality, motivation, charm, that are increasingly recognised as important for achieving adult social position^25^ and are therefore more socially mobile.

There is also an emphasis in both movements on the use of outdoor environments and physical activity.^12^ ^26^ Evidence suggests that one of the most important predictors of whether adults spend time in natural environments is whether they did so regularly as children.^27^ There is now evidence that exposure to natural outdoor environments is protective of mental health.^28^ Populations with greater exposure to natural environments have reduced socioeconomic inequalities in mental well-being,^29^ show cognitive benefits^30^ and better emotional well-being, with restorative experiences acting as the mediating factor.^31^ Physical activity is an important aspect of Scout–Guide practice, not premised on excellence at a particular activity^10^ but rather on participation. The benefits of physical activity for protecting or improving mental health are well established.^32^

Scouts–Guides use small groups to enable young people to learn about relationships, understand their own competences and to become more self-reliant.^23^ It may be that this early exposure to the skills needed to work with small group enables adults to more effectively develop later life social networks. Social interaction, in particular having friends, has been identified as being beneficial for emotional well-being for adults of a range of ages.^33^ It may also help individuals to engage with ‘local sociability’ and ‘community organisation’,^34^ if it exists in places they have lived, and thus benefit from informal reciprocity and social capital, more generally.

### Strengths and limitations of the study

This study used a prospective cohort design, controlling for many of the factors that might affect participation in Scouts–Guides and later life mental health. Most of these measures were collected in the appropriate cohort sweep. The possibility of residual confounding, particularly due to missing or poorly measured socioeconomic variables, was further examined using three methods, a negative exposure control, an exploration of ‘dose–response’ and a measure of differing regional rates of Scouts–Guide attendance. Although there was no evidence of a dose–response relationship, the other methods supported the plausibility of a causal relationship.

There were, however, some weaknesses in the study. Information on Scouts–Guide attendance was collected retrospectively, and there may therefore be recall bias. However, it seems unlikely that memory of attending would be associated with present mental health state. The negative exposure controls used were slightly non-standard. Typically, these are chosen because, a priori, there is no expectation they will be related to the outcome of interest, that they do is then taken as an indication of residual confounding. This would not be the case with voluntary group, church membership or other outside school clubs which might all, in theory, enhance social or other skills. However, they are starkly different from the structured and deliberate programme offered by Scouts–Guides, aimed at development. In this study, there was no positive relationship between the negative exposure controls and mental health, supporting the conclusion of no, attendance-related, common confounder. The dose–response measure was estimated from a question asked at aged 11 that may not necessarily relate to the period of attendance of scouts–guides. This may explain why there was no dose–response identified. In the absence of an instrumental variable, with exposure varying independently of any predictors of mental health, regional difference in participation in Scout–Guide troops was used. This measure will not definitely be exogenous with, for example, the possibility that a higher regional proportion of families and communities supporting the Scout–Guiding also creating an early life environment that was protective of later life mental health. However, for the Scout–Guide association with mental health to have been confounded by this ‘collective’ influence would require considerable variation to exist across the regions in the UK (because of the large variation in regional attendance levels). This seems implausible. While there were weaknesses in all four of the strategies used to assess whether non-causal associations might be confounding the relationship of interest, they all suggested residual confounding did not produce the positive association.

There were missing data necessitating the use of MI with its prerequisite assumptions. A large number of variables were used in the imputation model and analysis which could plausibly have affected the chance of not responding to a question. We also produced separate imputation models for those who had and had not been Scouts–Guides in the case of non-additive relationship with the key variable. The analysis was fully replicated for the complete case data set; we found no substantive differences between the two. We also conducted sensitivity analysis with the assumption that the data are MNAR. The results are consistent with those for the MAR assumption, suggesting the MAR assumption is reasonable (see online supplementary material). Sample attrition may have led to differential patterns of non-response between 1958 and 2008 (aged 50). However, analysis of attrition in the NCDS (by age 45) suggests that these study members are broadly representative of the surviving cohort and that the patterns of non-response were not differential with respect to the key variables analysed within our study.^14^

In this paper, we found evidence that participation in the Guides and Scouts in youth has a positive association with mental health some 40 years later in life. Crucially, the effect appeared particularly strong for children growing up in low social position households, ameliorating inequalities in later life probability of mental health based on childhood socioeconomic position. Given the relatively high cost to individuals and health systems of poor mental health in middle age, encouraging interventions in youth that are low cost and available worldwide through existing institutional structures may be an important and cost-effective policy response. It also suggests that future work could usefully investigate the long-term impact of other types of organised activities in childhood and youth.

**What is already known on this subject**

- Developmental youth programmes fostering: social, vocational and cognitive competence; self-confidence; connection to others and caring and compassion, are shown to have a positive effect on well-being in the short term.
- There is evidence that low or adverse socioeconomic status in childhood has deleterious effects on mental health in later life and social advantage is protective.
- There is little research on whether developmental youth programmes have a long-term impact on mental health.

**What this study adds**

- In this prospective birth cohort study of 9603, participation in Guides or Scouts was associated with significantly better mental health at age 50 and a reduction in adult mental health inequality associated with socioeconomic status position in childhood.
- Given the cost to health systems and individuals of poor mental health in adult life, interventions in youth, in particular those provided by volunteers, could be a cost-effective policy option.
