# Supplementary material for: Be(ing) prepared: Guide and Scout participation, childhood social position and mental health at age 50—a prospective birth cohort study
Source: J Epidemiol Community Health. 2016 Nov 10;71(3):275–81. doi: 10.1136/jech-2016-207898 (PMC5318648; doi:10.1136/jech-2016-207898)
Supplement: Supplementary data [file jech-2016-207898supp.pdf]

Online-Only Supplement

Missing values for 9603 cases were imputed using chained equations based on the following models:

SF-36 Mental Health Index	Truncated Regression
Father’s RGSC	Augmented Ordered Logistic Regression
Father’s CAMSIS	Truncated Regression
Family Difficulties - Mental Illness	Multinomial Logistic Regression
Parental hopes child's school leaving	Augmented Multinomial Logistic Regression
Play sport or go walking or swimming	Augmented Ordered Logistic Regression
Can walk 100 Yards	Augmented Logistic Regression
How often plays outdoor games & sport	Ordered Logistic Regression
How often plays indoor games & sport	Ordered Logistic Regression
Government Office Region at age 16	Ordered Logistic Regression
Pupil goes to clubs outside school	Ordered Logistic Regression

The covariates for the MI model were based the following variables (completed cases - all recorded at age 50):

Government Office Region
Highest Qualification attained
Frequency of Respondent exercising
How often Respondent visited friends in last two weeks
How often Respondent had friends visit in last two weeks
Church participation
Voluntary participation
Sex
Respondent NS-SEC
Number of Children
Marital Status.

Scout/Guide	Variable	Complete	Incomplete	Imputed	Total
Never	SF-36 Mental Health Index	6184	766	766	6950
	Father’s RGSC	6263	687	687	6950
	Father’s CAMSIS	4807	2143	2143	6950
	Family Difficulties - Mental Illness	6036	914	914	6950
	Parental hopes child's school leaving	5916	1034	1034	6950
	Play sport or go walking or swimming	6125	825	825	6950
	Can walk 100 Yards	6181	769	769	6950
	How often plays outdoor games & sport	4978	1972	1972	6950
	How often plays indoor games & sport	4898	2052	2052	6950
	Government Office Region at age 16	6319	631	631	6950
	Pupil goes to clubs outside school	5695	1255	1255	6950
Previous	SF-36 Mental Health Index	2441	212	212	2653
	Father’s RGSC	2418	235	235	2653
	Father’s CAMSIS	1905	748	748	2653
	Family Difficulties - Mental Illness	2364	289	289	2653
	Parental hopes child's school leaving	2300	353	353	2653
	Play sport or go walking or swimming	2432	221	221	2653
	Can walk 100 Yards	2440	213	213	2653
	How often plays outdoor games & sport	2011	642	642	2653
	How often plays indoor games & sport	1970	683	683	2653
	Government Office Region at age 16	2448	205	205	2653
	Pupil goes to clubs outside school	2277	376	376	2653
Overall	SF-36 Mental Health Index	8625	978	978	9603
	Father’s RGSC	8681	922	922	9603
	Father’s CAMSIS	6712	2891	2891	9603
	Family Difficulties - Mental Illness	8400	1203	1203	9603
	Parental hopes child's school leaving	8216	1387	1387	9603
	Play sport or go walking or swimming	8557	1046	1046	9603
	Can walk 100 Yards	8621	982	982	9603
	How often plays outdoor games & sport	6989	2614	2614	9603
	How often plays indoor games & sport	6868	2735	2735	9603
	Government Office Region at age 16	8767	836	836	9603
	Pupil goes to clubs outside school	7972	1631	1631	9603

Sensitivity analysis via pattern mixture approach with multiple imputation

The pattern-mixture method for sensitivity to the data being Missing Not At Random (MNAR) has been followed for two variables Father's CAMSIS and MHI-5 score.[1] For Father's CAMSIS, we drew 9603 observations from a normal distribution with a mean of -15 and a SD of 2.5 points and subtracted this from all missing cases that had been imputed. Where the subtracted score fell below 15, this was recoded as 15 as Father's CAMSIS is not observed below this range. For comparison, the figures are as follows (for the first of the 10 imputed datasets):

MAR| X:

CAMSIS Imputed Mean = 44.9

CAMSIS Imputed SD = 13.9

MNAR:

CAMSIS Prior Adjusted Mean = 33.3

CAMSIS Prior Adjusted SD = 15.8

For MHI-5, we drew 9603 observations from a normal distribution with a mean of -20 and a SD of 5 points and subtracted this from all missing cases that had been imputed. Where the subtracted score fell below 0, this was recoded as 0 as MHI-5 is not observed below this range. For comparison, the figures are as follows (for the first of the 10 imputed datasets):

MAR| X:

MHI-5 Imputed Mean = 74.9

MHI-5 Imputed SD = 18.2

MNAR:

MHI-5 Prior Adjusted Mean = 56.4

MHI-5 Prior Adjusted SD = 22.7

These are robust tests as it would be very unlikely that the non-respondents were so heavily skewed towards the lower end of the CAMSIS and MHI-5 distributions. The MAR model is robust to the distributional changes made when simulating MNAR conditions. The MAR model is reasonable and likely to be a conservative estimate of the Scout/Guide effect. The kernel density plots for the different distributions are shown on the next page. The model results are reported on the following page

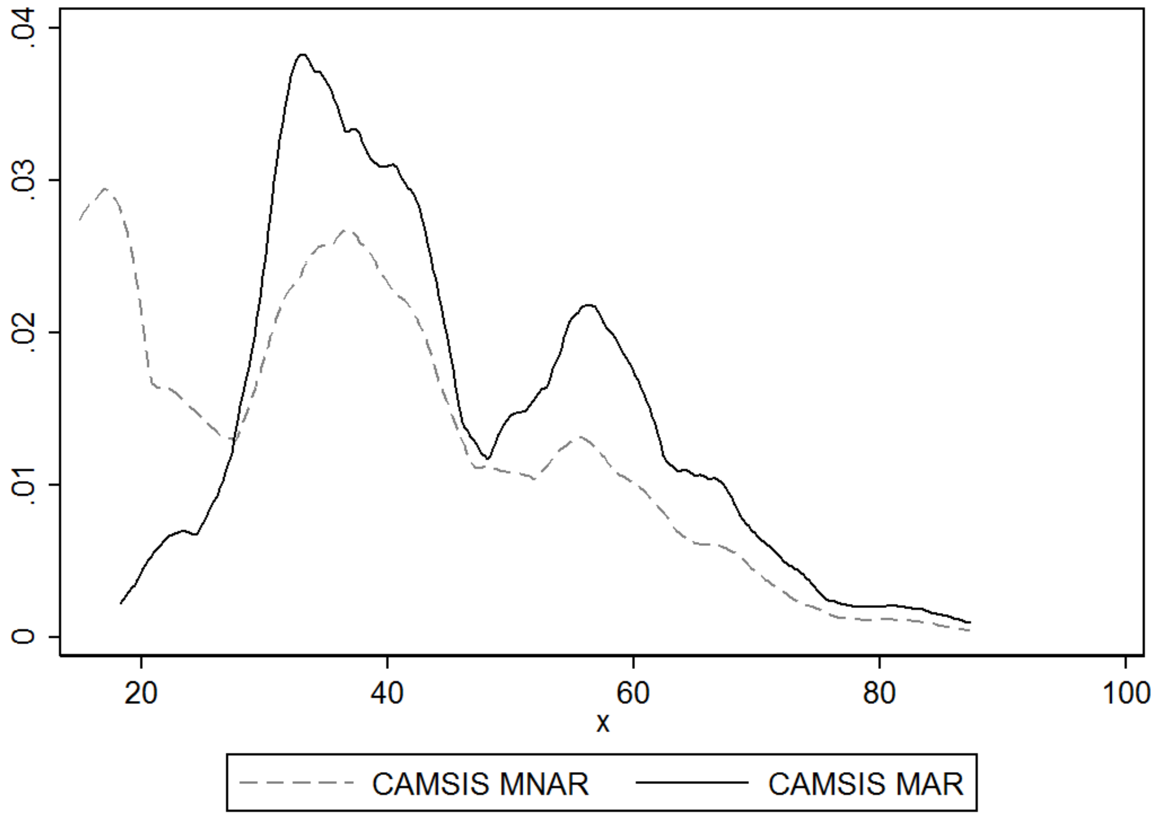
.04
.03
.02
.01
0
20
40
60
80
100
x
CAMSIS MNAR
CAMSIS MAR

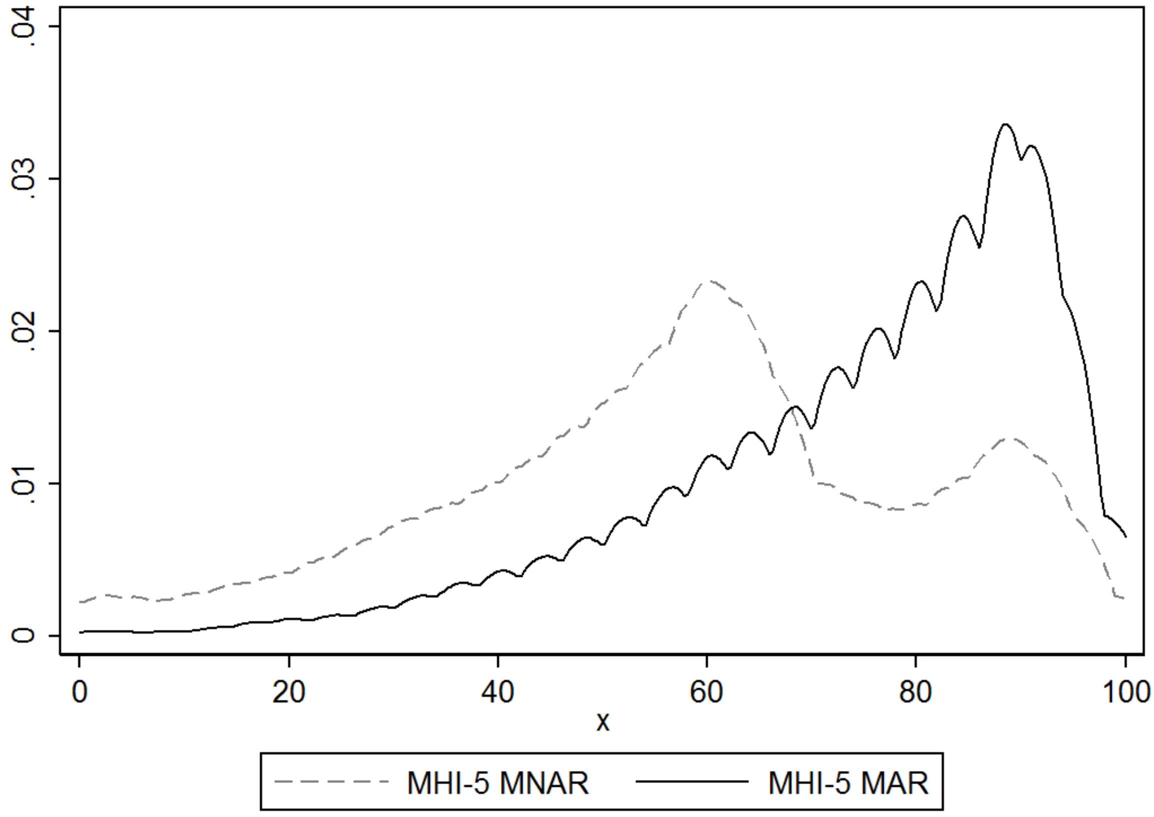
.04
.03
.02
.01
0
0
20
40
60
80
100
x
MHI-5 MNAR
MHI-5 MAR

	CC		MAR|X		CAMSIS		MHI-5		CAMSIS & MHI-5	
**Scout/Guide Participation (2008)**										
Never	0	0	0	0	0	0	0	0	0	0
Previous	2.56***	(0.62)	2.22***	(0.45)	2.21***	(0.45)	3.41***	(0.55)	2.08***	(0.51)
CAMSIS (Male Scale, based on Father's Occupation 1958)	0.028	(0.021)	0.060***	(0.016)	0.051***	(0.013)	0.081***	(0.020)	0.56***	(0.015)
**Voluntary Group Participation (2008)**										
Never	0	0	0	0	0	0	0	0	0	0
Previous	-0.97	(1.05)	-1.98**	(0.76)	-1.98**	(0.75)	-1.31	(0.93)	-2.07*	(0.90)
Current	-0.018	(1.35)	0.97	(0.96)	0.97	(0.96)	1.76	(1.18)	0.73	(1.11)
**Church Participation (2008)**										
Never	0	0	0	0	0	0	0	0	0	0
Previous	0.39	(1.01)	-0.60	(0.74)	-0.61	(0.74)	0.35	(0.90)	-0.66	(0.85)
Current	0.33	(0.98)	-0.18	(0.70)	-0.18	(0.70)	0.99	(0.87)	-1.10	(0.82)
**Sex (2008)**										
Female	0	0	0	0	0	0	0	0	0	0
Male	2.12***	(0.60)	1.70***	(0.40)	1.70***	(0.40)	1.70***	(0.50)	1.42**	(0.47)
**Family Difficulties - Mental Illness (1965)**										
No	0	0	0	0	0	0	0	0	0	0
Don't Know	-1.15	(1.13)	-0.34	(0.78)	-0.31	(0.78)	-1.74	(0.97)	-0.98	(0.93)
Yes	-6.33**	(2.11)	-5.47***	(1.30)	-5.40***	(1.31)	-8.37***	(1.53)	-6.34***	(1.50)
**Parental hopes child's school leaving (1969)**										
Leave min age	-5.29**	(1.61)	-5.79***	(0.96)	-5.77***	(0.96)	-9.10***	(1.19)	-4.76***	(1.10)
Stay on longer	0	0	0	0	0	0	0	0	0	0
Don’t know yet	-2.33**	(0.78)	-2.31***	(0.55)	-2.34***	(0.54)	-3.56***	(0.65)	-0.20	(0.60)
**How often plays outdoor games & sport (1974)**										
Often	4.01*	(1.75)	4.71**	(1.46)	4.73**	(1.46)	5.31**	(1.71)	3.82*	(1.57)
Sometimes	3.14	(1.72)	2.81	(1.46)	2.82	(1.46)	3.90*	(1.70)	2.22	(1.57)
Hardly ever	0.80	(1.75)	1.12	(1.45)	1.12	(1.45)	2.16	(1.75)	0.83	(1.62)
No chance	0	0	0	0	0	0	0	0	0	0
**How often plays indoor games & sport (1974)**										
Often	1.45	(1.09)	1.01	(0.88)	0.97	(0.88)	1.52	(1.06)	1.81*	(0.91)
Sometimes	-0.75	(1.03)	0.068	(0.78)	0.046	(0.78)	0.30	(0.94)	0.50	(0.83)
Hardly ever	-0.24	(1.04)	-0.013	(0.82)	-0.051	(0.82)	0.98	(1.06)	0.80	(0.94)
No chance	0	0	0	0	0	0	0	0	0	0
Constant	70.2***	(1.94)	68.4***	(1.61)	69.4***	(1.52)	47.6***	(1.91)	33.8***	(1.67)
Observations	4020		9603		9603		9603		9603	
R^2	0.03		0.03		0.03		0.04		0.18	

Online-Only Models Table 1: Linear Regression Models for Short Form Health Survey (SF-36) Mental Health Index (MHI-5) score.

**CC**	**Complete Cases model**
**MAR|X**	**Missing At Random Model (equivalent to model 2 in the paper)**
**CAMSIS**	**MNAR CAMSIS model**
**MHI-5**	**MNAR MHI-5 model**
**CAMSIS & MHI-5**	**MNAR CAMSIS & MHI-5 model**

	Model 5	
**Scout/Guide Participation**		
Never	0	0
Previous	3·50*	(1·57)
**CAMSIS**	0·067***	(0·018)
**Scout/Guide by CAMSIS Interaction**		
Never by CAMSIS	0	0
Previous by CAMSIS	-0·028	(0·033)
**Voluntary Group**		
Never	0	0
Previous	-1·96**	(0·75)
Current	0·97	(0·96)
**Church**		
Never	0	0
Previous	-0·60	(0·74)
Current	-0·20	(0·70)
**Sex**		
Female	0	0
Male	1·70***	(0·40)
**Family Difficulties - Mental Illness**		
No	0	0
Don't Know	-0·34	(0·78)
Yes	-5·49***	(1·30)
**Parental hopes child's school leaving**		
Leave min age	-5·77***	(0·97)
Stay on longer	0	0
Don’t know yet	-2·31***	(0·55)
**How often plays outdoor games & sport**		
Often	4·70**	(1·46)
Sometimes	2·81	(1·46)
Hardly ever	1·12	(1·45)
No chance	0	0
**How often plays indoor games & sport**		
Often	1·02	(0·87)
Sometimes	0·081	(0·78)
Hardly ever	-0·0052	(0·82)
No chance	0	0
Constant	68·0***	(1·61)
Observations	9603	
R^2	0·03	

eFull Models for Figure 1a: Linear Regression Models for Short Form Health Survey (SF-36) Mental Health Index (MHI-5) score

	Model 6	
**Scout/Guide**		
Never	0	0
Previous	2.32***	(0.63)
**RGSC: Social class of Mother's Husband**		
I	0.81	(1.11)
II	1.66*	(0.74)
III non-manual	1.80*	(0.82)
III manual	0	0
IV	0.79	(0.82)
V	-1.37	(0.89)
**Scout/Guide by RGSC Interaction**		
Never by I	0	0
Never by II	0	0
Never by III non-manual	0	0
Never by III manual	0	0
Never by IV	0	0
Never by V	0	0
Previous by I	-0.31	(1.94)
Previous by II	-0.59	(1.25)
Previous by III non-manual	-0.30	(1.45)
Previous by III manual	0	0
Previous by IV	-0.33	(1.53)
Previous by V	2.00	(1.75)
**Voluntary Group**		
Never	0	0
Previous	-1.93*	(0.75)
Current	1.02	(0.96)
**Church**		
Never	0	0
Previous	-0.57	(0.74)
Current	-0.12	(0.70)
**Sex**		
Female	0	0
Male	1.70***	(0.41)
**Family Difficulties - Mental Illness**		
No	0	0
Don't Know	-0.34	(0.77)
Yes	-5.46***	(1.30)
**Parental hopes child's school leaving**		
Leave min age	-5.90***	(0.97)
Stay on longer	0	0
Don’t know yet	-2.44***	(0.55)
**How often plays outdoor games & sport**		
Often	4.84**	(1.47)
Sometimes	2.95*	(1.47)
Hardly ever	1.21	(1.45)
No chance	0	0
**How often plays indoor games & sport**		
Often	0.97	(0.88)
Sometimes	0.044	(0.78)
Hardly ever	-0.029	(0.82)
No chance	0	0
Constant	70.5***	(1.51)
Observations	9603	
R^2	0.03	

Standard errors in parentheses, * p < 0·05, ** p < 0·01, *** p < 0·001

eFull Models for Figure 1b: Linear Regression Models for Short Form Health Survey (SF-36) Mental Health Index (MHI-5) score

	Model 7	
**Scout/Guide Participation (2008)**		
Never	0	0
Previous	2.29**	(0.71)
**Pupil goes to clubs outside school (1969)**		
Hardly ever	0	0
Sometimes	-0.30	(0.67)
Most days	0.076	(0.56)
**Scout/Guide by Pupil goes to clubs outside school (1969)**		
Never by Hardly ever	0	0
Never by Sometimes	0	0
Never by Most days	0	0
Previous by Hardly ever	0	0
Previous by Sometimes	0.32	(1.17)
Previous by Most days	-0.25	(1.12)
**RGSC: Social class of Mother's Husband (GRO 1951)**		
I	0.71	(0.92)
II	1.45*	(0.60)
III non-manual	1.71*	(0.67)
III manual	0	0
IV	0.72	(0.69)
V	-0.98	(0.81)
**Voluntary Group Participation (2008)**		
Never	0	0
Previous	-1.91*	(0.75)
Current	1.02	(0.96)
**Church Participation (2008)**		
Never	0	0
Previous	-0.57	(0.74)
Current	-0.12	(0.70)
**Sex (2008)**		
Female	0	0
Male	1.69***	(0.41)
**Family Difficulties - Mental Illness (1965)**		
No	0	0
Don't Know	-0.33	(0.78)
Yes	-5.48***	(1.30)
**Parental hopes child's school leaving (1969)**		
Leave min age	-5.92***	(0.97)
Stay on longer	0	0
Don’t know yet	-2.45***	(0.55)
**How often plays outdoor games & sport (1974)**		
Often	4.87**	(1.47)
Sometimes	2.98*	(1.47)
Hardly ever	1.22	(1.45)
No chance	0	0
**How often plays indoor games & sport (1974)**		
Often	0.96	(0.89)
Sometimes	0.043	(0.78)
Hardly ever	-0.034	(0.83)
No chance	0	0
Constant	70.6***	(1.48)
Observations	9603	
R^2	0·03	

Standard errors in parentheses, * p < 0·05, ** p < 0·01, *** p < 0·001

eFull Models for Figure 2: Linear Regression Models for Short Form Health Survey (SF-36) Mental Health Index (MHI-5) score

	All		Boys Only		Girls Only	
**Scout/Guide Participation (2008)**						
Never	0	(0)	0	(0)	0	(0)
Previous	2.22***	(0.45)	2.38***	(0.62)	2.08***	(0.62)
CAMSIS (Male Scale, based on Father's Occupation 1958)	0.060***	(0.016)	0.029	(0.022)	0.092***	(0.023)
**Voluntary Group Participation (2008)**						
Never	0	(0)	0	(0)	0	(0)
Previous	-1.98**	(0.76)	-3.31**	(1.24)	-1.22	(0.97)
Current	0.97	(0.96)	0.89	(1.47)	1.04	(1.28)
**Church Participation (2008)**						
Never	0	(0)	0	(0)	0	(0)
Previous	-0.60	(0.74)	-1.06	(1.13)	-0.21	(1.00)
Current	-0.18	(0.70)	-0.18	(1.11)	-0.12	(0.91)
**Sex (2008)**						
Female	0	(0)				
Male	1.70***	(0.40)				
**Family Difficulties - Mental Illness (1965)**						
No	0	(0)	0	(0)	0	(0)
Don't Know	-0.34	(0.78)	-0.70	(1.06)	0.028	(1.17)
Yes	-5.47***	(1.30)	-6.07***	(1.67)	-4.84*	(1.90)
**Parental hopes child's school leaving (1969)**						
Leave min age	-5.79***	(0.96)	-6.17***	(1.40)	-5.34***	(1.48)
Stay on longer	0	(0)	0	(0)	0	(0)
Don’t know yet	-2.31***	(0.55)	-2.58**	(0.77)	-2.06*	(0.79)
**How often plays outdoor games & sport (1974)**						
Often	4.71**	(1.46)	4.57	(2.61)	4.67**	(1.69)
Sometimes	2.81	(1.46)	2.82	(2.67)	2.73	(1.67)
Hardly ever	1.12	(1.45)	0.54	(2.77)	1.32	(1.70)
No chance	0	(0)	0	(0)	0	(0)
**How often plays indoor games & sport (1974)**						
Often	1.01	(0.88)	1.56	(1.25)	0.28	(1.30)
Sometimes	0.068	(0.78)	-0.14	(1.12)	0.27	(1.10)
Hardly ever	-0.013	(0.82)	-0.033	(1.27)	-0.040	(1.08)
No chance	0	(0)	0	(0)	0	(0)
Constant	68.4***	(1.61)	71.7***	(2.83)	66.8***	(2.14)
Observations	9603		4733		4870	
R^2	0.03		0.03		0.03	

Standard errors in parentheses, * $p < 0.05$, ** $p < 0.01$, *** $p < 0.001$

Online-Only Models Table 2: Linear Regression Models for Short Form Health Survey (SF-36) Mental Health Index (MHI-5) score. Sex-stratified analysis.

	All		Boys Only		Girls Only	
**Scout/Guide Participation (2008)**						
Never	0	(0)	0	(0)	0	(0)
Previous	2.22***	(0.45)	2.38***	(0.62)	2.08***	(0.62)
CAMSIS (Male Scale, based on Father's Occupation 1958)	0.060***	(0.016)	0.029	(0.022)	0.092***	(0.023)
**Voluntary Group Participation (2008)**						
Never	0	(0)	0	(0)	0	(0)
Previous	-1.98**	(0.76)	-3.31**	(1.24)	-1.22	(0.97)
Current	0.97	(0.96)	0.89	(1.47)	1.04	(1.28)
**Church Participation (2008)**						
Never	0	(0)	0	(0)	0	(0)
Previous	-0.60	(0.74)	-1.06	(1.13)	-0.21	(1.00)
Current	-0.18	(0.70)	-0.18	(1.11)	-0.12	(0.91)
**Sex (2008)**						
Female	0	(0)				
Male	1.70***	(0.40)				
**Family Difficulties - Mental Illness (1965)**						
No	0	(0)	0	(0)	0	(0)
Don't Know	-0.34	(0.78)	-0.70	(1.06)	0.028	(1.17)
Yes	-5.47***	(1.30)	-6.07***	(1.67)	-4.84*	(1.90)
**Parental hopes child's school leaving (1969)**						
Leave min age	-5.79***	(0.96)	-6.17***	(1.40)	-5.34***	(1.48)
Stay on longer	0	(0)	0	(0)	0	(0)
Don't know yet	-2.31***	(0.55)	-2.58**	(0.77)	-2.06*	(0.79)
**How often plays outdoor games & sport (1974)**						
Often	4.71**	(1.46)	4.57	(2.61)	4.67**	(1.69)
Sometimes	2.81	(1.46)	2.82	(2.67)	2.73	(1.67)
Hardly ever	1.12	(1.45)	0.54	(2.77)	1.32	(1.70)
No chance	0	(0)	0	(0)	0	(0)
**How often plays indoor games & sport (1974)**						
Often	1.01	(0.88)	1.56	(1.25)	0.28	(1.30)
Sometimes	0.068	(0.78)	-0.14	(1.12)	0.27	(1.10)
Hardly ever	-0.013	(0.82)	-0.033	(1.27)	-0.040	(1.08)
No chance	0	(0)	0	(0)	0	(0)
Constant	68.4***	(1.61)	71.7***	(2.83)	66.8***	(2.14)
Observations	9603		4733		4870	
R^2	0.03		0.03		0.03	

Standard errors in parentheses
* $p < 0.05$, ** $p < 0.01$, *** $p < 0.001$

Online-Only Models Sex stratified analysis

References

1. Carpenter JR, Kenward MG. *Multiple Imputation and its Application*. Hoboken, New Jersey: Wiley, 2013.
